# Color Characterization of Bordeaux Red Wines Produced without Added Sulfites

**DOI:** 10.3390/foods12122358

**Published:** 2023-06-13

**Authors:** Edouard Pelonnier-Magimel, Kléopatra Chira, Pierre-Louis Teissèdre, Michaël Jourdes, Jean-Christophe Barbe

**Affiliations:** 1 University of Bordeaux, Bordeaux INP, INRAE, OENO, UMR 1366, ISVV, F-33140 Villenave d’Ornon, France; epelonnier@gmail.com (E.P.-M.);; 2 Bordeaux Sciences Agro, Bordeaux INP, INRAE, OENO, UMR 1366, ISVV, F-33170 Gradignan, France

**Keywords:** wines without sulfites, ethylidene bridge, wine color, CIELab, polymeric pigments, UPLC-DAD/ESI QTof

## Abstract

Nowadays, the development of naturalness as a concept is illustrated in the oenological field by the development of wine produced with lower inputs, sometimes even without any addition of SO_2_ throughout the winemaking process, up to the bottling stage. Despite the increase in the offer of these wines, they remain poorly explored in the literature and require characterization. This study was developed to evaluate the color of Bordeaux red wines without SO_2_ addition using colorimetric and polymeric pigments analysis. From a batch of commercial Bordeaux red wines with and without SO_2_ addition, and experimental wines produced from homogenous grapes according to different winemaking processes, colorimetric analyses (CIELab and color intensity (CI)) revealed a large difference in wine color depending on the presence or absence of SO_2_. Indeed, wines without SO_2_ were significantly darker and presented with a deeper purplish color. According to these observations, polymeric pigments were quantified using UPLC-DAD/ESI QTof, and a higher concentration of polymeric pigments bound by the ethylidene bridge was observed in wines without SO_2._ This correlated with differences observed for CIELab and CI. Finally, a comparison with polymeric tannins bound by ethylidene bridge was made and revealed that no differences were observed between wines with and without added SO_2_. This underlines the affinity difference between tannins and anthocyanins to react with acetaldehyde to form ethylidene bridges.

## 1. Introduction

In the wine industry, sulfur dioxide (SO_2_) is currently the most used additive due to its antioxidasic [1], antimicrobial [2,3] and oxidation protection [4] properties. For wine preservation purposes, this additive is generally added throughout the winemaking process up to the bottling stage. SO_2_ has been commonly used in oenology since the 18th century [5], and historically it is supposed to have been used by the Romans for sanitizing wine vessels. Nowadays, the consciousness of the toxicity of SO_2_ [6], particularly for hypersensitive people, as well as the general evolution of consumers’ expectations [7] have lead to the development of wine with low input and, more particularly, wine without added SO_2_. In the European Community, Commission Regulation (EC) No 934/2019 formalizes the limits laid down by the International Organization of Vine and Wine (OIV). Currently, the maximum levels are, respectively 150 mg/L for red wine, 100 mg/L for organic red wine and 70 mg/L for certain biodynamic certifications. Finally, for wines produced without any added sulfites, the limit for total SO_2_ has been established at 10 mg/L, taking into account the small amount of this compound produced by yeast during alcoholic fermentation.

In red wines, the two main families of phenolic compounds are anthocyanins, at the origin of wine color, and condensed tannins, which are extracted from grapes’ skin and seeds; they contribute to the wine’s organoleptic quality due to their astringency and bitterness properties, as well as their role in the long-term color stability [8]. It is already known that condensed tannins influence the sensory perception of wine, but recently, interactions between anthocyanins and salivary proteins responsible for astringency perception were underlined [9,10]. Depending on the winemaking process, their content in wine ranged from 1 to 5 g/L. Structurally, anthocyanins are based on the flavylium (i.e., 2-phenylbenzopyrilium) ion having hydroxyl and methoxyl groups in different positions, as well as a glucose moiety esterified on the hydroxyl group at position 3. In *V. vinifera* grapes, the individual anthocyanins differ according to the substitution on their B-ring leading to the delphinidin, cyanidin, petunidin, peonidin and malvidin-3-*O*-glucosides. Each 3-*O*-glucoside forms can be acylated at the C6″ position of the glucose moiety by p-coumaric acid, caffeic acid or acetic acid [11,12]. On the other hand, condensed tannins are oligomers or polymers of flavanols formed by a benzopyran unit (rings A and C) with an aromatic cycle (ring B) linked to the carbon C-2 of the pyranic cycle (ring C). In grape seed and skin, the main monomers are (+)-catechin, (−)-epicatechin, (+)-gallocatechin, (−)-epigallocatechin and (−)-epicatechin-3-*O*-gallate [13]. In the oligomeric and polymeric structures of the condensed tannins, the monomeric flavanol units are linked together by B-type inter-flavan bonds between the carbon C4 of the upper unit and the carbons C8 or C6 of the lower unit. During red wine aging, both condensed tannins and anthocyanins undergo chemical changes leading to the formation of more stable polymeric pigments [14] through direct linkages [15] or indirect condensation, using acetaldehyde as an intermediate to form an ethylidene bridge between them [16]. Moreover, acetaldehyde can also react with the anthocyanin through a cycloaddition to form a pyranoanthocyanin such as vitisin B [17].

After alcoholic fermentation, acetaldehyde is also involved in a combination with free SO_2_ to form acetaldehyde–bisulfite, which is unable to react with condensed tannins and anthocyanins [18]. Thus, red wine produced without free SO_2_ will induce more free acetaldehyde to be involved in polyphenol polymerization. Regarding the impact of SO_2_ on organoleptic properties of red wine, in 2007, Gambuti et al. [19] described that the addition of SO_2_ before fermentation induced an increase in color intensity, color stability and the total concentration of phenolic compounds in Italian wines. These observations were explained by a higher extraction of phenolic compounds due to SO_2_ levels during maceration [20]. Moreover, as reported recently in Bordeaux red wines produced without added SO_2_ and without defects, a specific sensory space has been highlighted which was dependent on terroir and had a strong impact on mouth perception [21,22].

To date, the effect of SO_2_ on the wine color has not been exhaustively considered, except, mainly, its well-known direct role in anthocyanins discoloration. Thus, the aim of this study was to evaluate the wine color according to the occurrence of SO_2_ during the whole winemaking process and as well as over aging. The first approach was dedicated to spectrophotometric observations on commercial and experimental wines from different vintages produced with and without SO_2_ addition. Meanwhile, the second approach was based on the quantification of polymeric pigments, and especially those formed by indirect condensation, thanks to acetaldehyde, using an acidic depolymerization strategy [23] in order to better understand the impact of SO_2_ on the polyphenolic composition. Finally, determination of the analytical marker of ethylidene bridges between flaval-3-ol units was performed to evaluate the impact of acetaldehyde on polyphenolic polymerization.

## 2. Materials and Methods

### 2.1. Samples

Two sets of wines were analyzed. The first one was elaborated within IFV (Institut Français de la Vigne et du Vin) facilities, in Blanquefort (France) in 2017 and 2018. Grapes from Merlot R (*Vitis vinifera* L.) were harvested on the same plot and sampled to obtain homogenous batches. For each vintage, two maturity levels were studied (i.e., maturity A at technological maturity and maturity B harvested one week later) and then for each maturity level, two vinifications were performed (i.e., with and without SO_2_). Must analyses are presented in Appendix B Table A1. The first modality was close to usual practices using 50 mg/L of SO_2_ at vatting, and then maintaining the free SO_2_ level at 30 mg/L during aging and adding an extra 10 mg/L prior to bottling. The second modality was elaborated without any SO_2_ addition throughout the complete process. Alcoholic fermentations were managed in triplicate with inoculation of Active Dry Yeast *Saccharomyces cerevisiae* (Actiflore^®^ F33, Laffort, France, 200 mg/L). A commercial *Oenococcus oeni* preparation (Vitilactic^®^F1, Laffort, France, 10 mg/L) was used for malolactic fermentation. Wines were aged six months in stainless steel tanks, filtered (1 µm membrane), and blended and bottled in May 2018 and May 2019 for vintages 2017 and 2018, respectively.

The second set of wines samples consisted of Bordeaux commercial red wines from 2015 and 2016 vintages. This set was composed of 19 wines elaborated, according to their producers, without SO_2_ addition during the entire winemaking process and 16 wines produced with SO_2_. All the wines, with and without added SO_2_, were assayed, according to the Frantz-Paul method [24]. All of the studied wines without added SO_2_ presented a concentration of total SO_2_ under 30 mg/L, with a mean concentration of total SO_2_ at 4.0 ± 4.2 mg/L (mean ± standard deviation). Moreover, volatile acidity was evaluated with a WineScanTM Flex (FOSS Analytical, Hillerød, Denmark) in all wines without any statistical differences of volatile acidity between wines with and without added SO_2_, with a mean concentration at 0.41 ± 0.08 g/L expressed in acetic acid. The 16 wines with added SO_2_ were chosen to match wines without added SO_2_ (same varieties, same geographic origins and same prices). Thus, for 2015 vintage, 8 wines with and 8 wines without added SO_2_ were analyzed, whereas, for the 2016 vintage, analyses were conducted for 8 wines with and 11 wines without added SO_2_. All wines studied were evaluated without any sensory defect during blind tasting by an expert panel composed of 10 expert tasters on Bordeaux red wines defects.

### 2.2. Red Wine Colorimetric Analysis

#### 2.2.1. CIELab Analysis

CIELab parameters were evaluated using a Konica Minolta CM-5 spectrophotometer (Langenhagen, Germany) with a D65 light source and controlled by Spectramagic NX software (v.2.03). Calibration was performed before use, with transparency corresponding to 0% and black corresponding to 100%. To measure L*, a* and b* parameters [25], 10 mL of wine was filtered on 0.45 μm membrane. Measurements were performed in transparent glass cell with 1 cm of optical path. The color differences between two wines ΔE*ab were calculated as: $\Delta E^{*}\mathrm{ab}={\left[{\left(\Delta L^{*}\right)}^{2}+{\left(\Delta a^{*}\right)}^{2}+{\left(\Delta b^{*}\right)}^{2}\right]}^{1/2}$, with ΔL*, Δa* and Δb* from the determined value of the CIELab according to the International Organisation of Vine and Wine. For all samples, measurements were taken twice. L* represents lightness, the color coordinates a* and b* indicate the direction of colors: red to green (+a* towards red, −a* towards green) and yellow to blue (+b* towards yellow, −b* towards blue) [26]. Finally, chroma C* and hue angle h* were calculated as: $C^{*}={\left(a^{*}{}^{2}+b^{*}{}^{2}\;\right)}^{1/2}$ and $h^{*}=\arctan\left(b^{*}/a^{*}\right)$.

#### 2.2.2. Color Intensity Analysis

As a complement to CIELab analyses, spectrophotometric measures of the color intensity (CI) were also realized. Spectrophotometric analyses were performed using a Jasco V-630 spectrophotometer (Pfungstadt, Germany) using Spectra Manager (v. 2.09.01) software. For these analyses, all samples were measured twice. Chromatic parameters of wines, i.e., absorbances at 420 (d420), 520 (d520) and 620 nm (d620), were spectrophotometrically determined via a 1 mm optical path. The color intensity of the wine (CI) was determined by adding the optical densities at 420 (d420) and 520 nm (d520) [27] and the modified color density (CD) was obtained with the addition of absorbance at 620 nm (d620) to the two previous ones **[28]**, MilliQ water (resistivity: 18.2 MΩ cm, Milli-Q Plus water system, Millipore, Saint-Quentin-en-Yvelines, France) being used as a blank. Practically these absorbances were measured on a 1 mm optical path cell and multiplied by 10 to be expressed for a 1 cm cell **[29]**.

### 2.3. Total Phenolics Index

The total polyphenolic index (TPI) of each wine was measured after a 100-fold dilution with MilliQ water. Determinations were performed with a quartz cell of 1 cm of optical path at 280 nm cell. MilliQ water was used as a blank [29].

### 2.4. Polymeric Pigments Analyses by UPLC-DAD/ESI-QTof

#### 2.4.1. Fractionation

All wines were fractionated on a C-18 (octadecyl bonded, end-capped silica) cartridge (Supelco, St Quentin Fallavier, France). Next, 2.5 mL of wine was dried under reduced pressure and re-dissolved in 10 mL of MilliQ water, which was applied on the cartridge after conditioning with methanol (MeOH, analytical grade) from Prolabo-VWR (Fontenay sous Bois, France) and MilliQ water. The cartridge was eluted sequentially with 50 mL of MilliQ water, 50 mL of MeOH to collect the polyphenolic fraction. The polyphenolic fraction, eluted with MeOH, was dried under reduced pressure and re-dissolved in 1 mL MeOH.

#### 2.4.2. Acidic Depolymerization

The acidic depolymerization was performed as previously described by Zeng et al. (2016) and based on the phloroglucinolysis procedure [23]. This acidic depolymerization was performed twice for each wine studied. Indeed, the depolymerization reaction via phloroglucinolysis was largely used to calculate the mean degree of polymerization and led to the cleavage of carbon–carbon interflavonoid bound C4–C8 or C4–C6 [30]. In fact, this reaction allows us to specifically characterize polymeric pigments based on their specific quantification markers since only the B-type carbon–carbon bond between flavanol unit can be cleaved, and all the linkages between anthocyanins and flavanols (i.e., direct or indirect) are resistant to this acidic cleavage [23]. Phloroglucinolysis reagent solution containing 0.1 N HCl (Prolabo-VWR, Fontenays sous Bois, France) in methanol, 50 g/L phloroglucinol (Extrasynthese, Z.I Lyon Nord, France) and 10 g/L ascorbic acid (Prolabo-VWR, Fontenays sous Bois, France) was used for reaction. First, 200 μL of the MeOH fraction obtained after C-18 cartbridge was added to 200 μL of the phloroglucinolysis reagent solution. The reaction mixture was maintained at 50 °C for 20 min. Then, 1 mL of stop solution containing sodium acetate at 40 mmol/L (Merck KGaA, Darmstadt, Germany) in MilliQ water was added. Each reaction was performed in duplicate and was then analyzed by UPLC-DAD/ESI QTof after the end of the reaction.

#### 2.4.3. UPLC-DAD/ESI-QTof

The UPLC-DAD/ESI QTof system used was an Agilent 1290 (Agilent Technologies, Waldbronn, Germany) Infinity equipped with a diode-array detector (DAD) and an electrospray ionization (ESI)—QTof mass spectrometer (Agilent 6530 Accurate Mass, Agilent Technologies, Waldbronn, Germany). Chromatographic separation was carried out on an Eclipse Plus C18 column (2.1 mm × 100 mm, 1.8 μm). The solvents used were water (Optimal^®^ LC/MS, Fisher Scientific, Geel, Belgium) with 0.1% formic acid (Optimal^®^ LC/MS, Fisher Scientific, Geel, Belgium) for solvent A and MeOH (Optimal^®^ LC/MS, Fisher Scientific, Geel, Belgium) with 0.1% formic acid (Optimal^®^ LC/MS, Fisher Scientific, Geel, Belgium) for solvent B at low rate of 0.3 mL/min. Concerning polymeric pigments, 1 μL was injected and separated by a gradient of solvent B at 6% for 0.5 min; from 6 to 95% in 13.5 min; 95% for 4 min, and then the UPLC column was equilibrated for 3 min using the initial condition before the next injection **[23]**. The DAD signals were recorded at 280 nm and 520 nm. The ESI conditions were as follows: the gas temperature and flow were 350 °C and 9 L/min, respectively; the sheath gas temperature and flow were 350 °C and 11 L/min, respectively; the capillary voltage was 4000 V. All the analyses were performed in positive mode. All compounds present in Appendix B Table A2 were quantified in the equivalent of malvidin-3-*O*-glucoside. For 2,2′-ethylidenediphloroglucinol, the quantification marker of the ethylidene bridge between flavanol after phloroglucinolysis **[31]**, 1 μL was injected and separated by a gradient of solvent B at 6% during 0.5 min, from 6% to 50% in 19.5 min and from 50 to 100% in 5 min, and then the UPLC column was equilibrated for 2 min using the initial condition before the next injection. These analyses were performed in negative mode with same DAD and ESI conditions as those for polymeric pigments. Catechin, epicatechin, epigallocatechin, epicatechin-3-*O*-gallate and their adducts with phloroglucinol were quantified in the same conditions and allowed to determine the mean degree of polymerization (mDP).

### 2.5. Statistical Analysis

All statistical analyses were performed using RStudio software (v. 2023.03.0) (RStudio Inc., Boston, MA, USA, 2018). Tukey’s post hoc comparison test was performed when samples were significantly different after ANOVA, with a significance level of α = 5%. Moreover, Student’s parametric comparison test was also performed on data with significant level of α = 5% to independently evaluate data when the interaction between parameters was not significant. Pearson correlation tests were also used for correlation evaluation on parametric data.

## 3. Results and Discussion

### 3.1. Color Parameters

**Figure 1:**
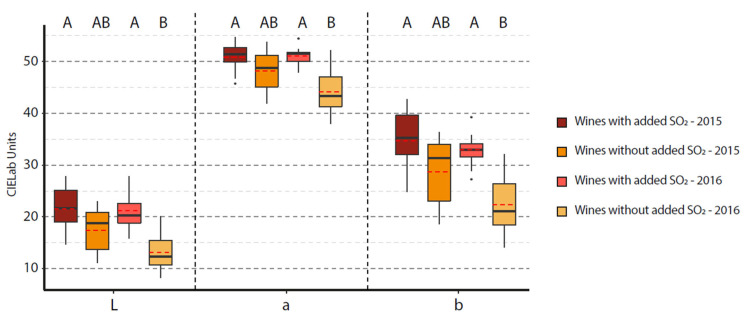
Boxplot of CIELab values for commercial wines with and without SO_2_ for 2015 and 2016 vintages. Dotted line represents mean value.

A colorimetric evaluation of experimental and commercial wines was undertaken with L*, a*, b* parameters and color intensity measurements. Figure 1 represents a boxplot of CIELab values for commercial wines. This boxplot shows that wines without added SO_2_ present L*, a* and b* values lower than the wines with added SO_2_, and that for the two vintages, wines from the 2016 vintage exhibited a statistically significant difference. Color differences ΔE*ab were calculated between wines with and without added SO_2_ for the two vintages. For the 2016 vintage, the wines presented a ΔE*ab at 14.70 ± 1.84 and for 2015 vintage a ΔE*ab at 7.93 ± 1.70. The human eye is able to perceive the difference for a ΔE*ab value higher than 2.7 [32]. These parameters revealed that wines without added SO_2_ were significantly darker, as described by the L* parameter, and presented a significant deeper violet color as described by a* and b* parameters. Inversely, wine with added SO_2_ present a significantly higher a*, associated with redder wines, than wines without added SO_2_. Moreover, C* and h* were calculated as previously described and follow observations made for L*, a* and b* values with significantly higher C* and h* values in wine with added SO_2_ compared to wines without added SO_2_ (Appendix B Table A3). These results underline an effective impact on the color for Bordeaux red wines produced without added SO_2_, which is linked to the use of this additive as previously observed in Aglianico wines [19]. Moreover, wines without added SO_2_ present a significantly higher CI (9.03 ± 1.7) value than wines with SO_2_ (6.77 ± 1.13) as well as higher CD, which is in agreement with the CIELab data that once again show the darker color of these wines. However, no significant differences were detected for TPI on these commercial wines, which suggests that the possible impact of SO_2_ on the polyphenol extraction during the winemaking process was not significatively observed with those wines. This is in contrast with the previously reported results. Nevertheless, to be sure of such an assessment, more specific analyses should have been performed.

Table 1 shows results concerning the same parameters for experimental wines. Concerning 2017 vintage at technological maturity, a high color difference ΔE*ab between wine with and without added SO_2_ was observed. Moreover, the former wine showed 50% higher color intensity. In addition, for the same vintage, at advanced maturity, wine without added SO_2_ presented L*, a* and b* values lower than for the wine with added SO_2_ as observed for technological maturity. Moreover, both CI and CD were higher for this wine than for the wine with added SO_2_. However, for this maturity level, color differences ΔE*ab between wines with and without added SO_2_ were lower than color differences ΔE*ab at technological maturity. Nevertheless, both corresponded to perceptible visual differences, wines without added SO_2_ being characterized by a darker deeper purplish color [33]. In parallel, the evaluation of wine color from the 2018 vintage revealed that CIELab parameters between wine with SO_2_ for 2017 and 2018 vintages follow a similar trend. Inversely, for wine without SO_2_, a large difference in CIELab parameters was observed, which suggests a possible higher oxidative evolution of wine color during aging in the bottle.

### 3.2. Quantification of Polymeric Pigments in Wines

**Figure 2:**
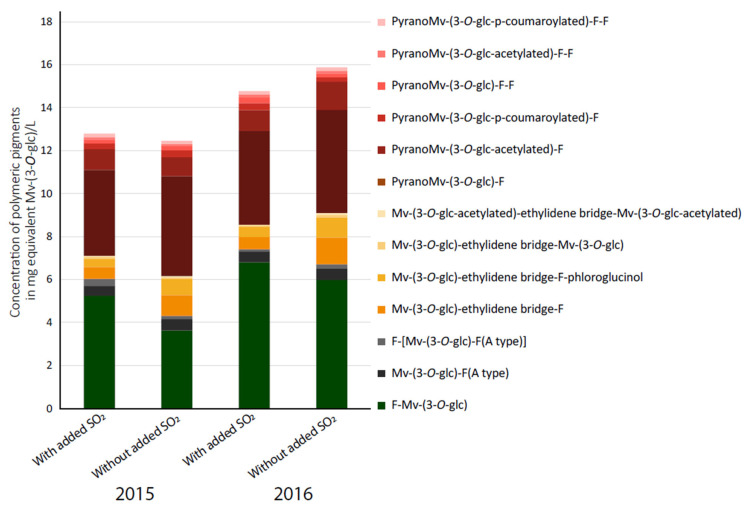
Total concentration of anthocyanin-derived pigments in commercial wines in mg equivalent malvidin-(3-*O*-glucose) per liter. Mv: malvidin, glc: glucose, F: flavan-3-ol.

Polymeric pigments corresponding to the polymerization of anthocyanins, especially malvidin-3-*O*-glucoside (Mv-3-*O*-glc) with condensed tannins (T), were quantified in all wines according to Zeng et al. (2016) [23]. All the known specific markers for each type of polymeric pigments released after acidic depolymerization were quantified, and Figure 2 shows the results for commercial wines. These released markers are mainly dimers of anthocyanins linked by direct or indirect linkages to flavanol (F), as well as their phloroglucinol adducts. In this figure, total concentrations by structural types are presented.

Firstly, these results revealed that total concentrations of polymeric pigments were almost similar between wines with and without added SO_2_ of the same vintage. Secondly, polymeric pigments T-Mv-(3-*O*-glc) and PyranoMv-(3-*O*-glc)-T prevailed in wines with and without added SO_2_. The third family of polymeric pigments measured was polymeric pigments bound by ethylidene bridges, especially structures Mv-(3-*O*-glc)-ethylidene bridge-T, which, for the 2015 vintage, showed a significantly higher concentration in wines without added SO_2_ (Figure 2). Indeed, during wine aging, monomeric anthocyanin concentrations decreased rapidly either through degradation or stabilization achieved via polymerization with tannins. In fact, alcoholic fermentation as well as oxidation during aging leads to acetaldehyde production which induces the formation of an ethylidene bridge between anthocyanins and condensed tannins. In the presence of free SO_2_, acetaldehyde takes carbonyl bisulfite form which cannot react with phenolic compounds [34]. In wines produced without any SO_2_ addition, free SO_2_ was not present; therefore, acetaldehyde was presented under its free form and could, thus, react with phenolic compounds, as observed in these wines. The same profile was observed for the 2016 vintage.

**Figure 3:**
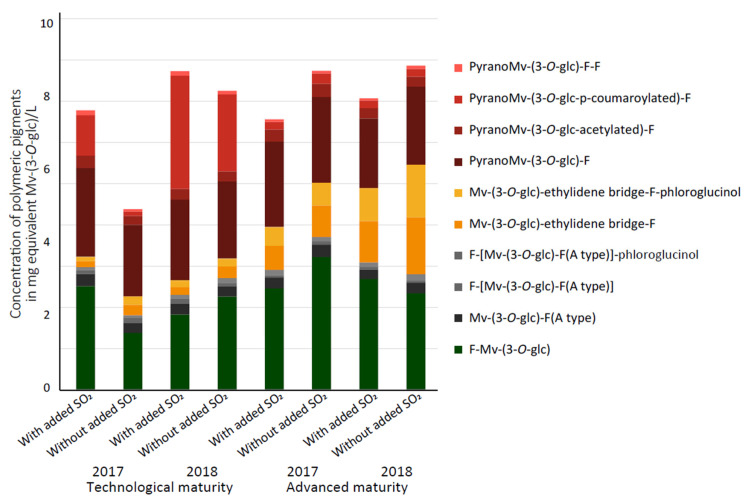
Total concentration of anthocyanin-derived pigments in experimental wines in mg equivalent malvidin-(3-*O*-glucose) per liter. Mv: malvidin, glc: glucose, F: flavan-3-ol.

Moreover, concerning experimental wines (Figure 3), the same behavior was observed with the majority of pigments with A-type linkage and PyranoMv-(3-*O*-glc)-T. For the same levels of maturity, higher concentrations of the ethylidene bridged pigments in wines without added SO_2_ were observed. Nevertheless, between the two vintages, an important difference in ethylidene bridged pigments was revealed. Indeed, for the two maturity levels, a higher concentration of these pigments was observed for the 2018 vintage compared to the 2017 vintage. In fact, wines from the 2018 vintage were analyzed a few months after their bottling, whereas wines from the 2017 vintage were analyzed after one year of aging in bottles. Even if it is not possible to draw conclusions about the origin of these lower ethylidene bridged pigments in wines which stood for a longer period in the bottle, these results seems in agreement with their possible oxidation such as, for example, that reported for the dimer Mv-(3-*O*-glc)-ethylidene bridge-F, which could be oxidized and produce a xanthylium-like derivative [35].

**Figure 4:**
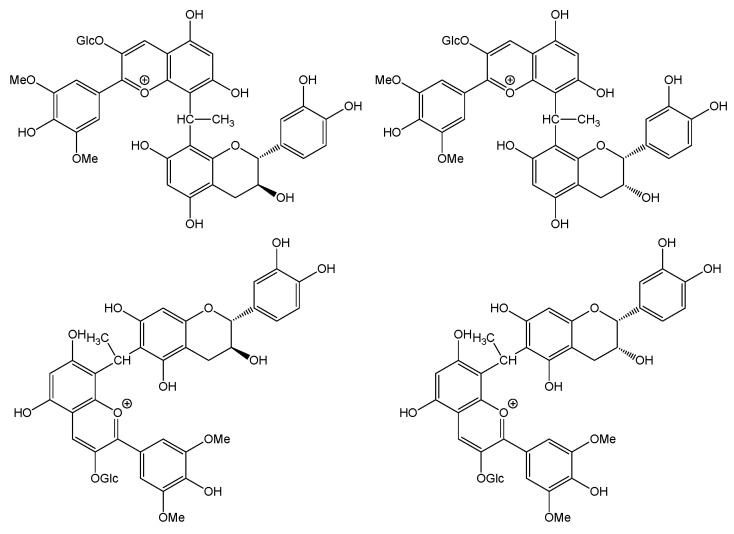
Structure of the four isomers of Mv-(3-*O*-glc)-ethylidene bridge-F.

At molecular level, for the dimer Mv-(3-*O*-glc)-ethylidene bridge-F, four isomers exist (Figure 4). Depending on the flavanol monomer, these will be epicatechin or catechin and, depending on the carbon involved in the inter-flavan bond, either C6 or C8 with the same *m*/*z* at 809.2287. Additionally, for the marker Mv-(3-*O*-glc)-ethylidene bridge-F-phloroglucinol obtained via acidic depolymerization of tannins moieties of the polymeric pigment formed by the Mv-(3-*O*-glc)-ethylidene bridge-F, only three isomers with *m*/*z* at 933.2448 are detected. Even if four isomers should be present, it is well known that the C6 of the catechin exhibits drastically lower reactivity compared to carbon C8 or carbon C6 of the epicatechin [36].

**Figure 5:**
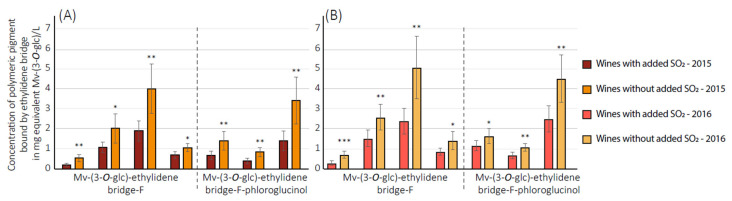
Concentration of polymeric pigment bound by ethylidene bridge in commercial wines in mg equivalent malvidin-(3-*O*-glucose) per liter. (**A**,**B**) correspond, respectively to 2015 and 2016 vintage. *** 0.1% significance, ** 1% significance, * 5% significance levels according Student comparison test, error bar represent confidence interval with a threshold of 0.05.

Figure 5 presents concentrations in the equivalent of malvidin-(3-*O*-glc) of the four dimers Mv-(3-*O*-glc)-ethylidene bridge-F and the three markers Mv-(3-*O*-glc)-ethylidene bridge-F-phloroglucinol for commercial wines from 2015 and 2016 vintages. As observed in this figure for all the above compounds, wines without added SO_2_ had a significantly higher concentration than wines with added SO_2_. This result confirmed the global observations of different evolutions of pigment polymerization between wines with and without added SO_2_ and highlighted an independent evolution of each pigment.

Moreover, considering standard error, it was possible to underline a higher dispersion of concentration found in wines without added SO_2_ compared to wine with. Indeed, in red wine, acetaldehyde may have four interaction types with phenolic compounds: (i) the production of ethylidene bridge between flavanols, (ii) the formation of oligomers anthocyanins and flavanols with ethylidene bridge, (iii) the formation of ethylidene bridge anthocyanin dimers and (iv) the production of vitisin B [37,38]. Moreover, the production of acetaldehyde during aging depends on oxidation and, consequently, on the impact of the oxygen exposure on wine composition. In wines with added SO_2_, the influence of the oxidative form of oxygen was probably more controlled and reduced by using SO_2_, which explain a lower oxidative evolution of the ethylidene linkage compounds and a lower variability between wines with added SO_2_. Nevertheless, in wine without added SO_2_, no difference was observed for the concentrations of anthocyanin transferred via the ethylidene bridge, which were less concentrated than the oligomer of anthocyanins transferred via the ethylidene bridge to condensed tannins. This fact underlined the higher reactivity of acetaldehyde for the formation of oligomer anthocyanins-condensed tannins than the polymerization of two anthocyanins. Moreover, in the absence of SO_2_, O_2_ intake could lead to oxidative reaction of wine compounds, notably ethanol to acetaldehyde, whereas, when free SO_2_ is present, this antioxidant will be oxidized instead of wine compounds. Thus, these differences in terms of oxygen level and availability could impact the color evolution over different lengths of time [39]. Ethylidene-linked pigments are usually known as being able to lead to further polycondensations of condensed tannins, which could increase the average size of the tannins [16]. Over time, this could affect more evolutions of these compounds [40] and could produce more complex oligomeric adducts [41]. It could also impact gustative properties; for instance, decreasing astringency [42].

**Figure 6:**
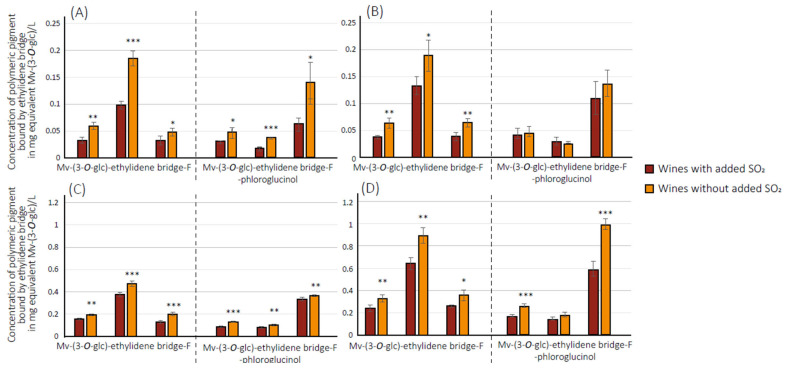
Concentration of polymeric pigment bound by ethylidene bridge in experimental wines in mg equivalent malvidin-(3-*O*-glucose) per liter. (**A**,**B**) correspond to 2017 vintage with, respectively, technological and advanced maturity and (**C**,**D**) correspond to 2018 vintage with, respectively, technological and advanced maturity. *** 0.1% significance, ** 1% significance, * 5% significance levels according Student’s comparison test, error bar represents confidence interval with a threshold of 0.05.

Globally, similar results for experimental wines were observed (Figure 6). However, only three out of four isomers of the dimer Mv-(3-*O*-glc)-ethylidene bridge-F have been detected. In commercial wines, the main isomer concentration was about 4 mg/L eq. Mv-(3-*O*-glc) in wine without added SO_2_ (2015 vintage), but in experimental wines, the maximum observed was about 1 mg/L eq. Mv(3-*O*-glc) in wine without added SO_2_ (2018 vintage). This important difference between the two sets of wines could explain the lack of detection of the minor isomer. However, as observed for commercial wines, a significantly higher concentration of these pigments was observed in wines without added SO_2_ than in wines with added SO_2_ for the two vintages and for the two maturity levels. This illustrates, as indicated above, the oxidative evolution of these newly formed pigments over time. Concerning the dimer form of two anthocyanins linked by the ethylidene bridge, this type of compound was not detected in experimental wines with and without added SO_2_. Although the same tendences were observed in experimental wines compared to commercial wines, the low levels of concentrations observed seem indicate that polymeric pigments bound by the ethylidene bridge may not explain colorimetric observations conducted for these wines and should implicate other mechanisms.

**Figure 7:**
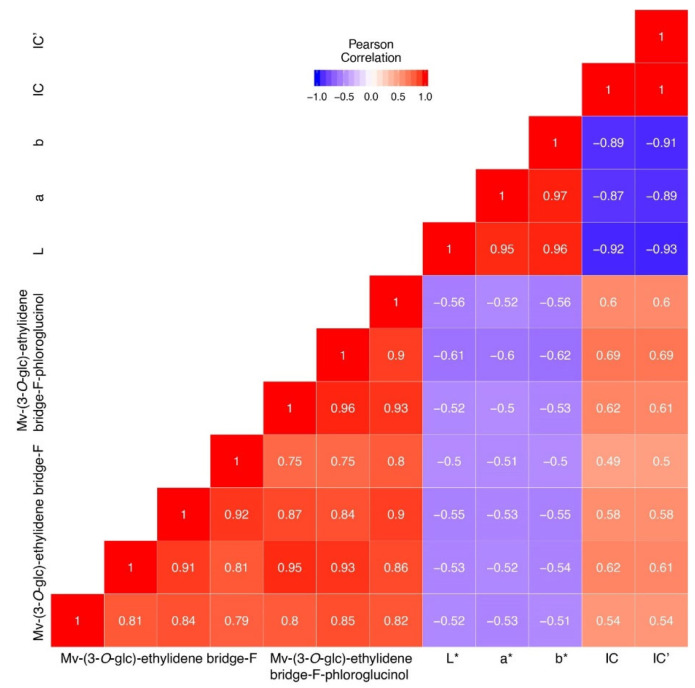
Heatmap of correlation coefficient of Pearson (r) for the seven oligomers bound by ethylidene bridge and spectrophotometric measure for commercial wines from the two vintages.

The change of color during aging from red–purple to brick red hues is attributed to the progressive formation of new pigments as anthocyanins react with other compounds and newly formed pigments undergo oxidative evolution. Pearson correlation tests were performed between quantified pigments and colorimetric values. As shown in the corresponding Figure 7, for commercial wines, CIELab parameters L*, a* and b* seem to be negatively correlated with the concentration of these pigments. Inversely, CI and CD seem to be correlated with the concentration of these pigments. The Pearson coefficient for these parameters was, respectively, between −0.5 and −0.62 for CIELab parameters and between 0.49 and 0.69 for polymeric pigments, which illustrated that polymeric pigments bound by ethylidene bridge concentrations were correlated with spectrophotometric observations. Indeed, it is already known that anthocyanins could react with SO_2_, leading to discoloration and, consequently, a decrease in CI and CD when SO_2_ is added [43]. Furthermore, negative correlation observed between CIELab parameters and polymeric pigments could be explained by the formation of an ethylidene bridge which develops purplish pigments associated with a decrease in a* and b* values. Previous studies [44,45] observed an augmentation of ethyl-linked pigment in model solution associated with an augmentation of CI and CD, which was also negatively correlated with an L* parameter corresponding to evolution towards a darker color.

### 3.3. Quantification of 2,2′-Ethylidenediphloroglucinol (EDP) in Wines

**Figure A1:**
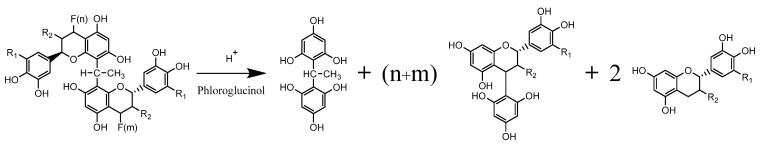
Reaction pathways of 2,2′-ethylidenediphloroglucinol, quantification marker of ethylidene bridge between flavan-3-ol.

In all commercial and experimental wines, EDP was quantified, since it is a specific marker of the incorporation of acetaldehyde into the tannins (reaction presented in Appendix A Figure A1). Indeed, the ethylidene bridge between flavanol units or between oligomers of condensed tannins, after phloroglucinolysis, leads to the formation of EDP. No significant difference was observed between wines produced with or without added SO_2_. This result highlights a difference in reactivity during the formation of ethylidene bridge between the formation of polymeric pigment and the tannins polymerization. More specifically, Mv-(3-*O*-glc) seems to present a higher affinity than catechin or epicatechin to react with epicatechin–ethanol or catechin–ethanol intermediates [45]. Moreover, in 2014, in synthesis conditions, Weber et al. [46] already underlined a higher formation of oligomeric structure anthocyanins–ethylidene bridge–tannins than dimeric structure tannins–ethylidene bridge–tannins. These data allow us to understand why EDP concentrations are similar in wines produced with or without SO_2_ and, consequently, why their mDP is similar.

## 4. Conclusions

Omitting the use of SO_2_ throughout the red wines winemaking process leads to the development of specific chromatic characteristics perceptible by the human eye. This color differentiation of wines without added SO_2_, which are darker and purplish compared to wines with added SO_2_, was correlated both with chromatic parameters and polymerization of polyphenolic compounds, especially formation of specific polymeric pigments involving ethylidene bridges, for the first time. This formation was linked to oxidation occurring during aging and led to acetaldehyde production from ethanol, with acetaldehyde being able to react to form ethylidene bridges when it is not bound with SO_2_. However, direct consequences for color have to be qualified, particularly considering the diversity of impacting compounds. Indeed, in wines without added SO_2_, free anthocyanins are not bound with SO_2,_ which is not the case for parts of them in wines with added SO_2_. This leads to color loss. Moreover, a differentiation of reactivity for polymerization through ethylidene bridges was highlighted, promoting the formation of polymeric tannins–ethylidene bridge–anthocyanins to the tannins–ethylidene bridge–tannins structure. This study is the first investigation to explore the chromatic characteristics of red wines without added SO_2_, with a specific focus on the formation of polymeric pigments, which are the key ones for color stabilization.

## Figures and Tables

**Table 1:** Values of spectrophotometric analysis for experimental wines with and without SO_2_ for 2017 and 2018 vintage.

			L*	a*	b*	CI	CD	ΔE*ab
2017	Technological maturity	With SO_2_	16.75	46.45	27.88	0.643	0.729	17.62
		Without SO_2_	8.64	38.05	14.68	0.937	1.087	
	Advanced maturity	With SO_2_	8.66	38.35	14.73	0.999	1.085	4.22
		Without SO_2_	7.06	35.60	11.95	1.071	1.238	
2018	Technological maturity	With SO_2_	17.51	48.39	28.88	0.715	0.817	4.81
		Without SO_2_	15.50	45.65	25.48	0.713	0.823	
	Advanced maturity	With SO_2_	12.46	43.30	21.22	0.887	1.023	2.44
		Without SO_2_	13.71	44.49	22.99	0.676	0.805	

## Data Availability

The data presented in this study are available on request from the corresponding author.

## Sample Availability

Samples of the compounds are available from the authors.

## Appendix B

**Table A1:** Sugar, total acidity and pH values of must from experimental wines for the two vintages and maturity levels.

	Sugar (g/L)	Total Acidity (g/L H_2_SO_4_)	pH
Without SO_2_ Technological maturity 2017	222	3.10	3.45
With SO_2_ Technological maturity 2017	222	3.10	3.45
Without SO_2_ Advanced maturity 2017	234	2.70	3.58
With SO_2_ Advanced maturity 2017	234	2.70	3.58
Without SO_2_ Technological maturity 2018	216	2.80	3.57
With SO_2_ Technological maturity 2018	216	2.80	3.57
Without SO_2_ Advanced maturity 2018	219	2.65	3.61
With SO_2_ Advanced maturity 2018	219	2.65	3.61

**Table A2:** Polymeric pigments and quantification markers studied in wines. Mv: malvidin, glc: glucose, F: flavan-3-ol. From Zeng et al.(2016) and Zeng (2015) [23,47].

No.	Name	Molecular Formula [M]^+^	*m/z*	Retention Time (min)
P1	F-Mv-(3-*O*-glc)	[C_38_H_37_O_18_]^+^	781.1974	4.720
P2				5.404
P3				6.679
P4				7.172
P5	Mv-(3-*O*-glc)-F(A type)	[C_38_H_37_O_18_]^+^	783.2131	6.690
P6				7.514
P11	F-(Mv-(3-*O*-glc)-F(Atype))-phloroglucinol	[C_44_H_41_O_18_]^+^	907.2291	5.490
P12				5.648
P13	Mv-(3-*O*-glc)-ethylidene bridge-F	[C_40_H_41_O_18_]^+^	809.2287	7.839
P14				7.980
P15				8.263
P16				8.396
P17	Mv-(3-*O*-glc)-ethylidene bridge-F-phloroglucinol	[C_46_H_45_O_21_]^+^	933.2448	7.562
P18				7.845
P19				8.053
P21	Mv-(3-*O*-glc)-ethylidene bridge- Mv-(3-*O*-glc)	[C_48_H_51_O_24_]^+^	1011.2765	9.536
P22				11.209
P25	Mv-(3-*O*-glc-acetylated)-ethylidene bridge- Mv-(3-*O*-glc-acetylated)	[C_52_H_55_O_26_]^+^	1095.2976	11.028
P26				12.886
P42	PyranoMv-(3-*O*-glc)-F	[C_40_H_37_O_18_]^+^	805.1974	9.336
P43				9.886
P44	PyranoMv-(3-*O*-glc-acetylated)-F	[C_42_H_39_O_19_]^+^	847.2080	9.611
P45				9.819
P46	PyranoMv-(3-*O*-glc-p-coumaroylated)-F	[C_49_H_43_O_20_]^+^	951.2342	9.769
P47	PyranoMv-(3-*O*-glc-)-F-F	[C_45_H_49_O_24_]^+^	1093.2610	7.353
P48				7.436
P49	PyranoMv-(3-*O*-glc-acetylated)-F-F	[C_57_H_51_O_25_]^+^	1135.2714	7.561
P50				7.644
P51	PyranoMv-(3-*O*-glc-p-coumaroylated)-F-F	[C_64_H_55_O_26_]^+^	1239.2976	8.468
P52				8.468
P53				8.468
P54				8.468

**Table A3:** Mean CIELab values for commercial wines with and without added SO_2_ for 2015 and 2016 vintages. SD: Standard deviation.

Vintage	Wine	L*		a*		b*		C*		h*	
		Mean	SD	Mean	SD	Mean	SD	Mean	SD	Mean	SD
2015	With added SO_2_	21.38	4.66	50.88	2.99	34.71	6.26	61.69	5.84	0.59	0.06
	Without added SO_2_	15.91	4.80	46.72	4.56	26.56	7.25	53.87	7.53	0.50	0.03
2016	With added SO_2_	20.13	3.91	50.82	2.17	32.33	4.21	60.26	3.92	0.56	0.07
	Without added SO_2_	12.16	2.52	43.37	3.11	20.71	3.9	48.12	4.61	0.44	0.05
